# MM-129 as a Novel Inhibitor Targeting PI3K/AKT/mTOR and PD-L1 in Colorectal Cancer

**DOI:** 10.3390/cancers13133203

**Published:** 2021-06-26

**Authors:** Justyna Magdalena Hermanowicz, Krystyna Pawlak, Beata Sieklucka, Robert Czarnomysy, Iwona Kwiatkowska, Adam Kazberuk, Arkadiusz Surazynski, Mariusz Mojzych, Dariusz Pawlak

**Affiliations:** 1 Department of Pharmacodynamics, Medical University of Bialystok, Mickiewicza 2C, 15-222 Bialystok, Poland; beata.sieklucka@umb.edu.pl (B.S.); iwona.kwiatkowska@umb.edu.pl (I.K.); dariuszpawlak@poczta.onet.pl (D.P.); 2 Department of Clinical Pharmacy, Medical University of Bialystok, Mickiewicza 2C, 15-222 Bialystok, Poland; 3 Department of Monitored Pharmacotherapy, Medical University of Bialystok, Mickiewicza 2C, 15-222 Bialystok, Poland; krystyna.pawlak@umb.edu.pl; 4 Department of Synthesis and Technology of Drugs, Medical University of Bialystok, Mickiewicza 2C, 15-222 Bialystok, Poland; robert.czarnomysy@umb.edu.pl; 5 Department of Medicinal Chemistry, Medical University of Bialystok, Mickiewicza 2C, 15-222 Bialystok, Poland; adam.kazberuk@umb.edu.pl (A.K.); arkadiusz.surazynski@umb.edu.pl (A.S.); 6 Department of Chemistry, Siedlce University of Natural Sciences and Humanities, 3 Maja 54, 08-110 Siedlce, Poland; mariusz.mojzych@uph.edu.pl

**Keywords:** 1,2,4-triazine derivative, Akt—protein kinase B, PD-L1—programmed death ligand 1, xenograft, colon cancer

## Abstract

**Simple Summary:**

MM-129 (1,2,4-triazine derivative) is a novel promising drug candidate against colon cancer. It has the ability to inhibit intracellular pathways promoting tumorigenesis with a simultaneous reduction of PD-L1 expression, a key element of the cancer immune escape axis. MM-129 may also act as a chemosensitizer, overcoming chemoresistance against 5-FU, the first-line agent in the chemother-apy of colon cancer. Our results significantly expand knowledge and help better understand the process of tumorigenesis, the intracellular pathways involved, and the mutual interactions of in-dividual proteins, and create the possibility of their pharmacological blockade. There is a real chance that the obtained results and the conclusions drawn on their basis will help in the development of a new, effective therapy, which could be an attractive alternative to the already existing methods of colon cancer treatment.

**Abstract:**

Background and aims: The purpose of the present study was to examine the pharmacodynamics features of MM-129 (1,2,4-triazine derivative) as a novel promising drug candidate against colon cancer. Methods: MM-129 was assessed for antitumor activity through an in vivo study on Cby.Cg-Foxn1nu/cmdb mice. The mechanistic studies investigated cellular affinity of a new 1,2,4-triazine derivative by measuring levels of intracellular/extracellular signal molecules participating in tumorigenesis. Results: The results revealed that MM-129 significantly reduced tumor growth in mice challenged with DLD-1 and HT-29 cells. It exerted the ability to inhibit intracellular molecules promoting tumorigenesis and inducing cell cycle arrest, like Akt, mTOR, and CDK2. Simultaneously, it was able to downregulate PD-L1 expression, which involves immunological self-tolerance. Combined administration of MM-129 and 5-fluorouracil (5-FU) additionally amplified these effects, which were manifest as an increase population of cells in the G0/G1 phase. Conclusions: A novel 1,2,4-triazine derivative with a dual mechanism of antitumor activity—MM-129, may act as a chemosensitizer, overcoming chemoresistance against 5-FU, the first-line agent in the chemotherapy of colon cancer.

## 1. Introduction

The continuing growth rate of colorectal carcinoma incidence indicates a necessity for intensified research and forces us to seek newer methods for early detection and more ideal treatment of patients suffering from this type of cancer. The currently used therapy is undeniably unsatisfactory. The entire oncological world is eagerly awaiting the next substances that will significantly improve treatment effectiveness, positively affect prognosis and patients’ life expectancy, and improve quality of life. Triazine is a chemical species of six-membered heterocyclic ring compound possessing three nitrogen in its structure, with the general formula C3H3N3. The names of the three isomers indicate which of the carbon-hydrogen units on the benzene ring position of the molecule have been replaced by so-called 1,2,3-triazine, 1,2,4-triazine, and 1,3,5-triazine, respectively [1]. Compounds containing the 1,2,4-triazine moiety are studied intensively due to their broad range of biological activities, such as antifungal, anti-inflammatory, analgesic, anticancer and many others [1,2,3,4,5]. Recently, the novel 1,2,4-triazole derivatives have been implicated in mediating antitumor activities. These compounds have inhibitory effects on the growth of several types of cultured cancer cells, including leukemia, and breast and colorectal cancer [6,7,8].

MM-129 (pyrazolo[4,3-e]tetrazolo[4,5-b][1,2,4]triazine sulfonamide) is a chemical compound obtained by chemical synthesis, with a similar chemical structure to roscovitine, which is in the clinical trial phase (NCT00999401). Our previous in vitro observations have clearly shown that MM-129 is more effective than the hitherto used preparations, including the reference drug 5-fluorouracil. It statistically significantly reduces cell viability and inhibits DNA biosynthesis of DLD-1 and HT-29 colorectal cancer cell [9]. The phosphoinositide-3-kinase (PI3K)/AKT/mTOR pathway plays a key role in the regulation of processes related to cell growth, metabolism, survival, and proliferation. An increased expression of this kinase is observed in numerous cancers, including lung, prostate, breast, pancreatic, and colorectal [10]. Recent studies have indicated an association of the PI3K/Akt pathway with colon tumorigenesis. The activation of Akt signaling has been observed in 60–70% of human colon cancers, and inhibitors of PI3K/Akt signaling have been indicated as potential therapeutic agents [11].

Cyclin-dependent kinases (CDKs) are enzymes essential for cell-cycle progression. They can be divided into two groups, including ‘cell cycle’ CDKs, which directly regulate cell-cycle transitions and cell division, and ‘transcriptional’ CDKs, which mediate gene transcription [12,13]. Cyclin-dependent kinase 2 (CDK2) is a serine/threonine protein kinase, which has a role in the G1/S transition, the initiation of DNA synthesis, and the regulation of the exit from S phase [14,15]. Its activity is controlled by a tumor suppressor p53 in a p21Cip-dependent mechanism. A low level of functional p53 in different types of cancer, including colon, lung, breast, brain, prostate, ovary, and liver, was observed [16].

Programmed death ligand 1 (PD-L1), also known as B7 homolog 1 and CD274, is a type I transmembrane glycoprotein involved in the establishment and/or maintenance of immunological self-tolerance by suppressing T-lymphocyte activity, proliferation, and effector functions. PD-L1 expression was detected on cell surfaces of numerous types of cancer, including kidney, lung, ovarian, bladder, and melanoma [17,18,19,20]. The presence of this protein has also been demonstrated in colorectal carcinoma cells [21]. Clinical trials data clearly indicate that high PD-L1 expression directly correlates with cancer metastasis and stage, and worse prognosis [22,23,24].

The goal of the present study was to evaluate the pharmacodynamics features of MM-129, a new 1,2,4-triazine derivative, as a promising drug candidate against colon cancer. To investigate the cellular mechanism underlying the anticancer activity of this novel agent, we assessed the impact of MM-129 on specific cellular and molecular targets, including PD-L1 expression.

## 2. Materials and Methods

### 2.1. Establishment of Xenograft

All procedures were performed in accordance with the guidelines for animal experiments and the protocol approved by the Local Ethical Committee on Animal Testing (Permit No. 92/2019). Animal studies are reported in compliance with the ARRIVE guidelines [25]. Experiments were conducted on four/six-week-old mice, weighing 18–20 g, inbred strain Cby.Cg-Foxn1nu/cmdb, Centre of Experimental Medicine, Poland. The characterization of strain, breeding conditions, and experimental protocol were described previously [26]. Tumor volumes based on caliper survey were calculated using the modified ellipsoidal formula [27]:

$$V=\frac{\pi }{6}\;f\;{\left(lenght\times width\right)}^{\frac{3}{2}}$$

According to tumor volume measurements and mouse weight, the mice were allocated into four treatment groups for DLD-1 xenografts (*n* = 10) and four treatment groups for HT-29 xenografts (*n* = 10). When the tumors reached a diameter of about 5 mm, that is, according to the literature the size suitable to conduct further phases of research, the intraperitoneal administration of MM-129 (2.5 mg/kg), 5-FU (50 mg/kg), or their combination was started [28]. The dose was chosen based on our preliminary study, in which we assessed the anticancer activity of MM-129 [9,29]. The control group was administered with MM-129 solvent (10% DMSO/PBS). The treatment was continued for two weeks. Animals were euthanized by an overdose of pentobarbital administered intraperitoneally.

### 2.2. Cell Cultures

DLD-1 (ATCC, Cat# CCL-221, RRID:CVCL_0248) and HT-29 (ATCC, Cat# HTB-38, RRID:CVCL_0320) cell lines of human colorectal adenocarcinoma were obtained from the American Type Culture Collection (ATCC, Manassas, VA, USA). Characteristic cell lines were presented earlier [26].

### 2.3. Microscope Bioimaging

Cells were plated in 96-well culture plates optimized for imaging applications at 1 × 10^4^ cells per well. After the treatment with 5-FU at concentration 50 µM, MM-129 (1 µM, 10 µM, and 100 µM) and a combination of these drugs (5-FU 50 µM + MM-129 10 µM) the cells were fixed with a 3.7% formaldehyde solution at room temperature for 15 min and permeabilized with a 0.1% Triton X-100 solution at room temperature for 10 min. Then non-specific binding was blocked (3% BSA). After that time, the cells were rinsed, incubated with rabbit polyclonal Ser 473 antibody against phospho Akt1/2/3 (Thermofisher, Cat# PA5-104445), rabbit polyclonal antibody against phospho mTOR (Thermofisher Cat# 44-1125G), mouse monoclonal antibody against phospho ERK1/2 (Thermofisher Cat# 14-9109-82), mouse monoclonal antibody against phospho p53 (Thermofisher Cat #MA5-15229), rabbit polyclonal antibody against phospho CDK2 (Abcam Cat# ab-68265), and mouse monoclonal antibody against PD-L1 (Abcam Cat# ab238697) for 1 h at room temperature. Then, the cells were rinsed and incubated with an FITC-conjugated secondary goat polyclonal antibody against mouse (Sigma-Aldrich, Cat# F0257) or a secondary goat polyclonal antibody against rabbit (Sigma-Aldrich, Cat# A3687) for 60 min in the dark. After washing, the nuclei were stained with Hoechst 33342 (2 μg/mL). Cells were analyzed using a confocal microscope BD Pathway 855 using a 20× objective.

### 2.4. Western Blot

DLD-1 and HT-29 cells were incubated (24 h) with 5-FU at concentration 50 µM, MM-129 (1 µM, 10 µM and 100 µM), and a combination of these drugs (5-FU 50 µM + MM-129 10 µM). Western blotting was performed using a standard method described previously [27]. The sample used for electrophoresis consisted of 20 μg of protein from 6 pooled cell extracts from separate experiments (*n* = 6). Three independent gels were run for each examined protein, and densitometry of band staining was used for statistical analysis. The nitrocellulose was incubated with rabbit polyclonal Ser 473 antibody against phospho Akt1/2/3 (Thermofisher, Cat# PA5-104445), rabbit monoclonal H-136 antibody against Akt1/2/3 (Santa Cruz Biotechnology, Cat# sc-8312), rabbit polyclonal antibody against phospho mTOR (Thermofisher Cat# 44-1125G), rabbit polyclonal antibody against mTOR (Thermofisher Cat# PA5-34663), mouse monoclonal antibody against phospho ERK1/2 (Thermofisher Cat# 14-9109-82), mouse monoclonal antibody against ERK1/2 (Thermofisher Cat# 14-9108-82), mouse monoclonal antibody against phospho p53 (Thermofisher Cat #MA5-15229), mouse monoclonal antibody against p53 (Abcam Cat #ab16465), rabbit polyclonal antibody against phospho CDK2 (Abcam Cat# ab-68265), mouse monoclonal antibody against CDK2 (Thermofisher Cat# MA5-17052), mouse monoclonal antibody against PD-L1 (Abcam Cat# ab238697), or mouse monoclonal antibody against beta-actin (Sigma-Aldrich, Cat# A2228) in TBS-T (20 mM Tris-HCl buffer (pH 7.4) containing 150 mM NaCl and 0.05% Tween 20) overnight. HRP secondary goat polyclonal antibody against mouse (Thermofisher, Cat# G-21040) or HRP secondary goat polyclonal antibody against rabbit (Thermofisher, Cat# G-21234) was added at a 1:5000 dilution in TBS-T and incubated for 1 h with slow shaking. After washing in TBS-T (4 × 10 min), the membranes were incubated with Amersham ECL Western Blotting Detection Reagent, (GE Healthcare Life Sciences, Little Chalfont, Buckinghamshire, UK). Pictures were taken using the BioSpectrum Imaging System UVP (Ultra-Violet Products Ltd., Cambridge, UK).

### 2.5. Flow Cytometry

Cell cycle analysis of DLD-1 and HT-29 was performed by the use of the fluorescence image cytometer FACSCanto II flow cytometer (BD Bioscences Systems). The colon cancer cells were cultured with 5-FU at concentration 50 µM, MM-129 (1 µM, 10 µM, and 100 µM), and a combination of these drugs (5-FU 50 µM + MM-129 10 µM) for 24 h. Cells were trypsinized and fixed with cold ethanol (70%) at 4 °C and stored overnight at −20 °C. After removing ethanol, the cells were washed three times with PBS, treated with 50 μg/mL of DNase-free RNase A Solution (Promega), and stained with 100 μg/mL of PI.

### 2.6. Quantitative-Real-Time-PCR (QRT-PCR)

Total RNA was isolated from cell cultures using Total RNA Mini Plus (A&A Biotechnology, Poland), following the manufacturer’s protocol. Quality control and concentration measurement of isolated RNA was performed using the Thermo Scientific NanoDrop 2000 spectrophotometer. Reverse transcription was performed with iScript™ Reverse Transcription Supermix (BioRad, Irvine, CA, USA), according to the manufacturer’s instructions (RNA input was 0.8 μg). Quantitative real-time PCR (QRT-PCR) was performed using the Stratagene Mx3005P QPCR System (Agilent Technologies, Santa Clara, CA, USA) and SsoAdvanced™ Universal SYBR^®^ Green Supermix (BioRad, USA). The samples were prepared as duplicates and contained 10.0 ng of cDNA and 300.0 nM of primers mix. PrimePCR™ PCR Primers were obtained from BioRad (BioRad, USA). Reference numbers of primers are: qHsaCED0003497 (CDK2), qHsaCID0011338 (AKT), qHsaCID0013658 (p53, TP53), qHsaCID0036468 (PD-L1), qHsaCED0038674 (GAPDH). The final volume of the reaction was 10 μL per well. The thermal cycling profile was set as follows: 30 sec in 95 °C for polymerase activation and DNA denaturation, then 40 cycles of 10 sec in 95 °C, and 30 sec in 60 °C for the denaturation and extension steps. The fluorescence level was measured at the end of each cycle. The quality of the amplified products was determined by the analysis of melting curves (65–95 °C, 0.5 °C increments at 2 sec/step). In addition, no-RT, no-primers samples were run to ensure that only a single product was amplified. The results of gene expression for the genes of interests were normalized to the expression of the housekeeping gene, which was GAPDH. Relative gene expression was analyzed by the comparison of Ct values using the ΔΔCt method.

### 2.7. Statistical Analysis

Shapiro–Wilk’s W test of normality was used for data distribution analysis. The normally distributed data were analyzed using a one-way analysis of variance (ANOVA) and shown as mean ± SD. The non-Gaussian data were presented as median (full range) and analyzed using the non-parametric Kruskal–Wallis test. Statistical analysis was conducted using GraphPad Prism 7.4 software (GraphPad Prism 7.04 Software, USA). The differences were deemed statistically significant when *p* < 0.05. Quantifications of Western blots were analyzed using UPV instrument.

## 3. Results

### 3.1. MM-129 Has Beneficial Effects in Eliminating Colon Cancer

**Figure 1:**
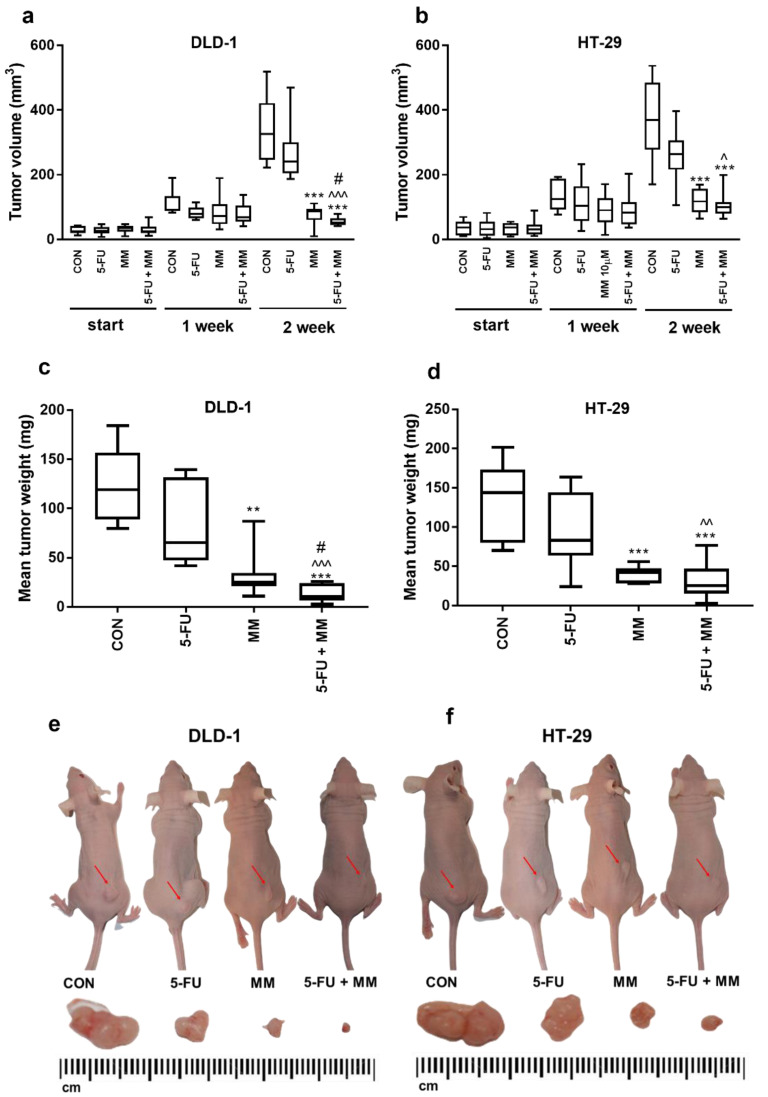
Impact of MM-129 (MM 2.5 mg/kg), 5-FU (50 mg/kg), or a combination of these agents (5-FU + MM) on tumor volume and mean tumor weight in DLD-1 (**a**,**c**,**e**) and HT-29 (**b**,**d**,**f**) xenografts. Start—before agent administration; 1, 2—after first and second week of treatment. Data presented as median with range, *n* = 10. ** *p* < 0.01, *** *p* < 0.001 vs. CON, ^ *p* < 0.05, ^^ *p* < 0.01, ^^^ *p* < 0.001 vs. 5-FU, ^#^ *p* < 0.05 vs. MM in the second week of experiment.

The antitumor activity of MM-129 was examined in a mouse model of xenotransplantation (Figure 1). During the whole experiment, animal welfare was ensured. First, mice were injected subcutaneously on the dorsal side with 50 µL of suspension containing 1 × 10^8^ DLD-1 or HT-29 cells in PBS, according to the method described by Shinohara et al. [30]. Five days after cell injection, solid tumors were established in 80 mice (100% of animals). In DLD-1 xenografts as well as in HT-29 xenografts, tumor growth was significantly inhibited in the group receiving MM-129 compared with the control in the second week (Figure 1). Despite the difference in tumor size reported at the start in the DLD-1 group treated with MM-129 (35.1 (9–46) mm^3^) compared with the control (25.0 (12–43) mm^3^), the significant decrease of tumor development is worthy of attention. In the second week of the experiment, we observed significant inhibition of tumor growth in the MM-129-treated group in DLD-1 (to 26.5% of control; *p* < 0.001) and in HT-29 xenografts (to 32% of control; *p* < 0.001) (Figure 1a,b). Incubation with MM-129 + 5-FU significantly intensified tumor volume reduction in both DLD-1 (to 52.6 (41–79) mm^3^) and HT-29 xenografts (to 100.9 (63–199) mm^3^) compared with the appropriate controls: 325.6 (222–518) mm^3^ and 369.2 (170–536) mm^3^, respectively, both *p* < 0.001. The combination of MM-129 + 5-FU showed more pronounced anticancer activity than 5-FU alone in DLD-1 (240.0 (186–469] mm^3^; *p* < 0.001) and HT-29 cells (263.9 (105–396) mm^3^; *p* < 0.05). Similarly, the combination of these compounds was also more effective in reducing cancer cells than MM-129 alone in DLD-1 (86.6 (9*–*111) mm^3^; *p* < 0.05). A stronger anticancer effect was observed in DLD-1 xenografts, leading to the reduction of tumor weight by about 91% compared to 82% in HT-29 xenografts. These results indicate that MM-129 showed a more pronounced anticancer effect when used in combination with 5-FU, particularly in DLD-1 xenografts.

### 3.2. MM-129 Inhibits the Intracellular Tumor-Promoting Pathway

**Figure 2:**
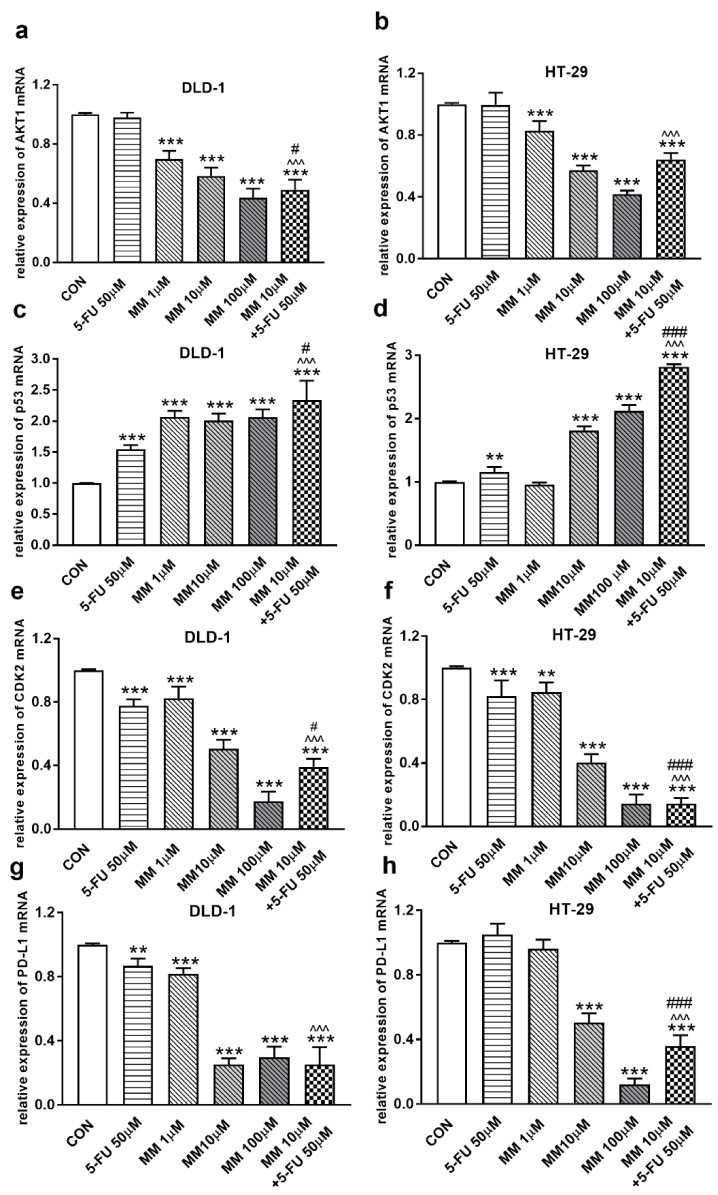
Akt1 (**a**,**b**), p53 (**c**,**d**), CDK2 (**e**,**f**), and PD-L1 (**g**,**h**) mRNA levels in DLD-1 and HT-29 cells after 24 h incubation with 5-FU (5-FU 50 µM), MM-129 (MM 1 µM, 10 µM, 100 µM), and their combination (MM 10 µM + 5-FU 50 µM). The results are presented as means ± SDs, *n* = 6. ** *p* < 0.01, *** *p* < 0.001 vs. CON, ^^^ *p* < 0.001 vs. 5-FU, ^#^ *p* < 0.05, ^###^ *p* < 0.001 vs. MM-129 at dose 10 µM.

**Figure 3:**
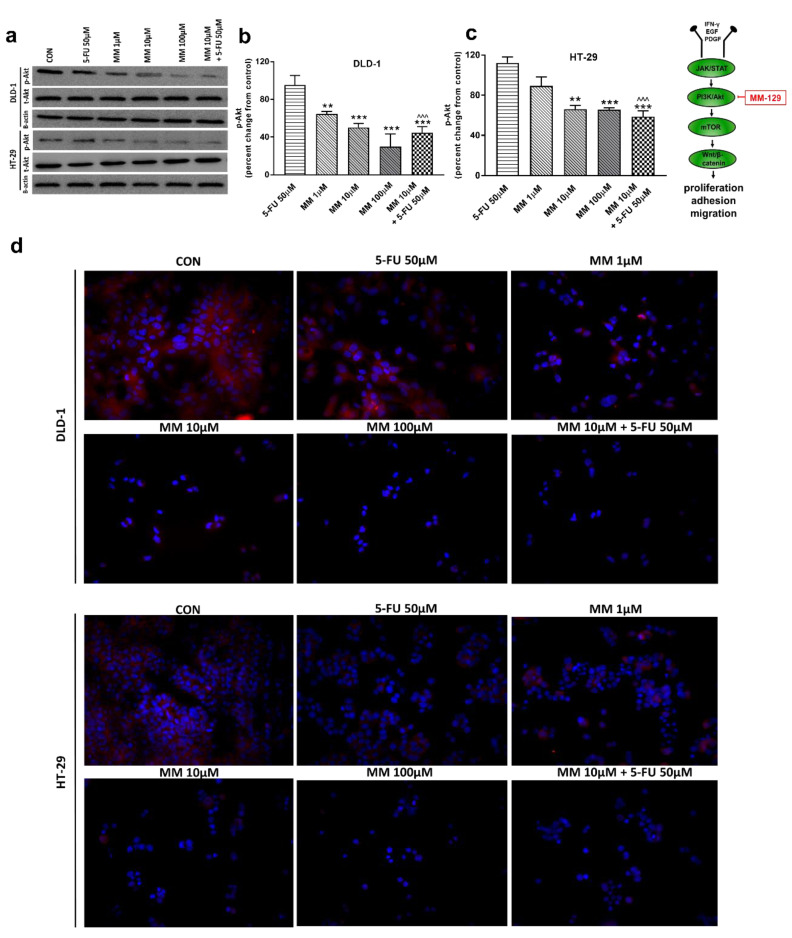
Phosphorylated Akt 1/2/3 (p-Akt), total Akt 1/2/3 (t-Akt), and β-actin expression as determined by Western blot (**a**) and phosphorylated Akt (p-Akt) determined by confocal microscopy (**d**) in DLD-1 and HT-29 cells treated with 5-FU (5-FU 50 µM), MM-129 (MM 1 µM, 10 µM, 100 µM), and their combination (MM 10 µM + 5-FU 50 µM) for 24 h. The samples used for electrophoresis consisted of 20 µg of protein from 6 pooled cell extracts from independent experiments (*n* = 6). Band staining was quantified by densitometry (**b**,**c**). The corresponding uncropped blots are shown in Supplementary Figures S1a–c and S2a–c. Cells were incubated with rabbit polyclonal Ser 473 antibody against phospho Akt1/2/3 and secondary goat polyclonal antibody against rabbit (red label). The nuclei were stained with Hoechst 33342 (blue label) (**d**). The results are presented as means ± SDs; * *p* < 0.05, ** *p* < 0.01, *** *p* < 0.001 vs. CON, ^^^ *p* < 0.001 vs. 5-FU.

**Figure 4:**
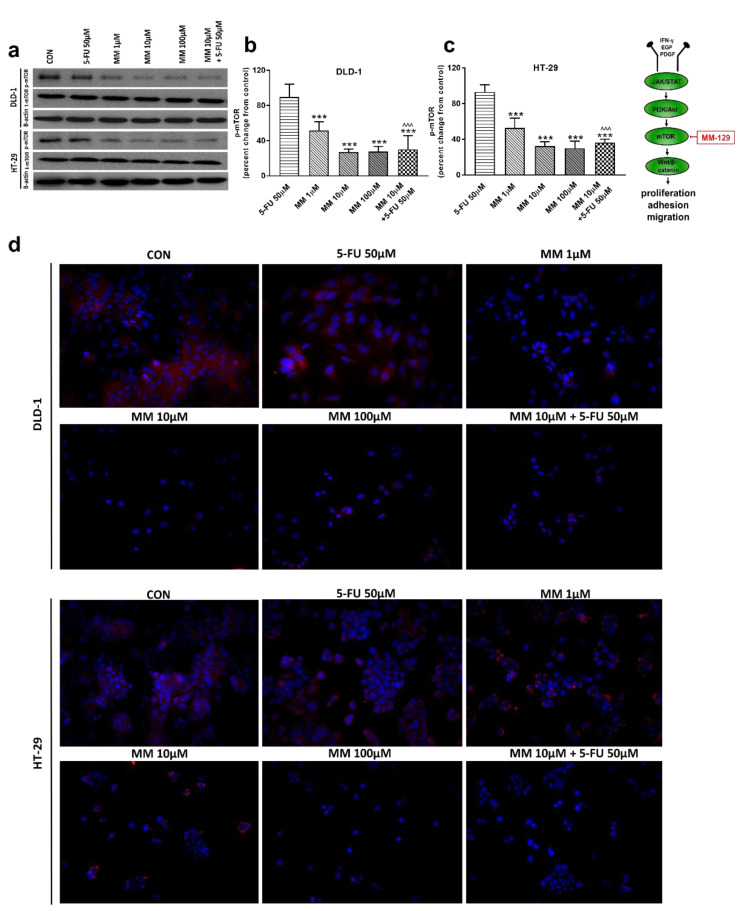
Phosphorylated mTOR (p-mTOR), total mTOR (t-mTOR), and β-actin expression as determined by Western blot (**a**) and phosphorylated mTOR (p-mTOR) determined by confocal microscopy (**d**) in DLD-1 and HT-29 cells treated with 5-FU (5-FU 50 µM), MM-129 (MM 1 µM, 10 µM, 100 µM), and their combination (MM 10 µM + 5-FU 50 µM) for 24 h. The samples used for electrophoresis consisted of 20 µg of protein from 6 pooled cell extracts. The samples used for electrophoresis consisted of 20 µg of protein from 6 pooled cell extracts from independent experiments (*n* = 6). Band staining was quantified by densitometry (**b**,**c**). The corresponding uncropped blots are shown in Supplementary Figures S3a–c and S4a–c. Cells were incubated with rabbit polyclonal antibody against phospho mTOR and secondary goat polyclonal antibody against rabbit (red label). The nuclei were stained with Hoechst 33342 (blue label) (**d**). The results are presented as means ± SDs; *** *p* < 0.001 vs. CON, ^^^ *p* < 0.001 vs. 5-FU.

**Figure 5:**
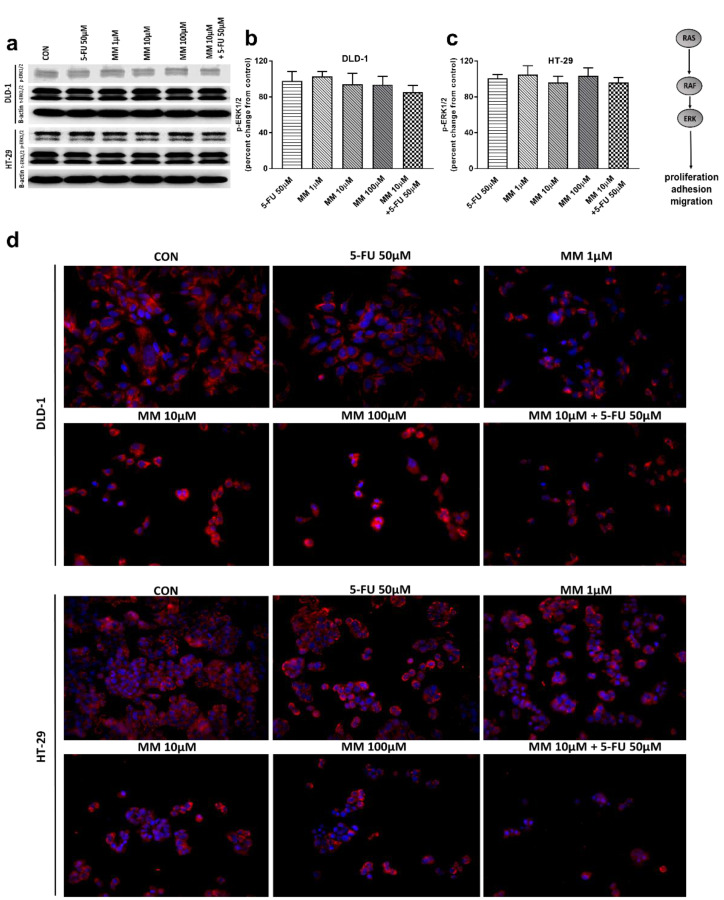
Phosphorylated ERK1/2 (p-ERK1/2), total ERK1/2 (t-ERK1/2), and β-actin expression as determined by Western blot (**a**) and phosphorylated ERK1/2 (p-ERK1/2) determined by confocal microscopy (**d**) in DLD-1 and HT-29 cells treated with 5-FU (5-FU 50 µM), MM-129 (MM 1 µM, 10 µM, 100 µM), and their combination (MM 10 µM + 5-FU 50 µM) for 24 h. The samples used for electrophoresis consisted of 20 µg of protein from 6 pooled cell extracts. The samples used for electrophoresis consisted of 20 µg of protein from 6 pooled cell extracts from independent experiments (*n* = 6). Band staining was quantified by densitometry (**b**,**c**). The corresponding uncropped blots are shown in Supplementary Figures S5a–c and S6a–c. Cells were incubated with mouse monoclonal antibody against phospho ERK1/2 and FITC conjugated secondary goat polyclonal antibody against mouse (red label). The nuclei were stained with Hoechst 33342 (blue label) (**d**). The results are presented as means ± SDs.

To investigate the mechanisms mediating the anticancer effects of MM-129, the expression and levels of intracellular proteins, such as Akt, mTOR, and ERK1/2, were determined by RT-PCR, Western blot, and confocal microscopy (Figure 2, Figure 3, Figure 4 and Figure 5). Akt, a serine/threonine protein kinase, also called protein kinase B, is the main signal transducer of the PI3K/Akt/mTOR pathway. Increased Akt kinase activity is not usually a sufficient factor responsible for initiating the oncogenesis process, but it contributes to tumor progression by inhibiting apoptosis, and promoting proliferation, migration, and invasion [10].

It has been also presented that Akt activation was closely associated with chemoresistance in colon cancer, and its inhibition may overcome 5-FU-resistance in SNU-C5/5-FU cells [31,32]. The incubation of DLD-1 and HT-29 cells with MM-129 downregulated in dose-dependent manner Akt mRNA expression compared with the control (Figure 2a,b). RT-PCR analysis also showed that a simultaneous use of 5-FU + MM-129 downregulated Akt mRNA expression compared with 5-FU alone (in both cell lines) and compared with MM-129 alone (in DLD-1 cells). Using Western blot, we found a decrease of p-Akt expression after MM-129 stimulation at all tested doses compared with the control in DLD-1, and at 10 µM and 100 µM in HT-29 (Figure 3a–c). 5-FU + MM-129 caused the strongest reduction in the p-Akt level compared with 5-FU, particularly in DLD-1 cells. Similar results were observed using bioimaging microscopy. A high level of phosphorylated Akt (p-Akt) was indicated in the control cells, in which the whole nuclei (Hoechst, blue) and the recruitment of p-Akt (red) were stained (Figure 3d). Exposure to MM-129 as well as 5-FU + MM-129 resulted in downregulated p-Akt expression compared with the control and 5-FU in both DLD-1 and HT-29. These results clearly indicate that MM-129 exhibits antitumor activity in the Akt-dependent mechanism.

In a further step, we assessed the mTOR protein expression, which is a critical regulator of cellular metabolism, growth, and proliferation. It is well known that the mechanism of mTOR regulation takes place through the activation of the PI3K/Akt pathway; however, mTOR also receives input from multiple signaling pathways [33]. Western blot and confocal microscopy showed decreased p-mTOR levels after incubation with MM-129, starting from just 1 µM concentration. p-mTOR levels dropped below control values also after 5-FU + MM-129 treatment in both lines (Figure 4a–d). In turn, this effect was not observed after DLD-1 and HT-29 incubation with 5-FU.

Extracellular signal-related kinases (ERKs) act as pleiotropic molecules in tumors, where they activate pro-survival pathways, leading to cell proliferation and migration, as well as modulate apoptosis, differentiation, and senescence [34]. Mitogens and growth factors use the Ras/Raf/MEK/ERK pathway to modulate gene expression and inhibit apoptosis [35]. MM-129, 5-FU, and the combination of 5-FU with MM-129 did not change the p-ERK1/2 level compared with control in both DLD-1 and HT-29 (Figure 5a–c). Confocal microscopy also confirmed that both MM-129 and 5-FU did not change the ERK1/2 levels (Figure 5d). These results indicate that the new 1,2,4-triazine derivative does not affect ERK1/2-dependent signaling in the tumor cells analyzed in this study.

### 3.3. MM-129 Evokes Cell Cycle Arrest via Upregulation of p53 and Downregulation of CDK2

**Figure 6:**
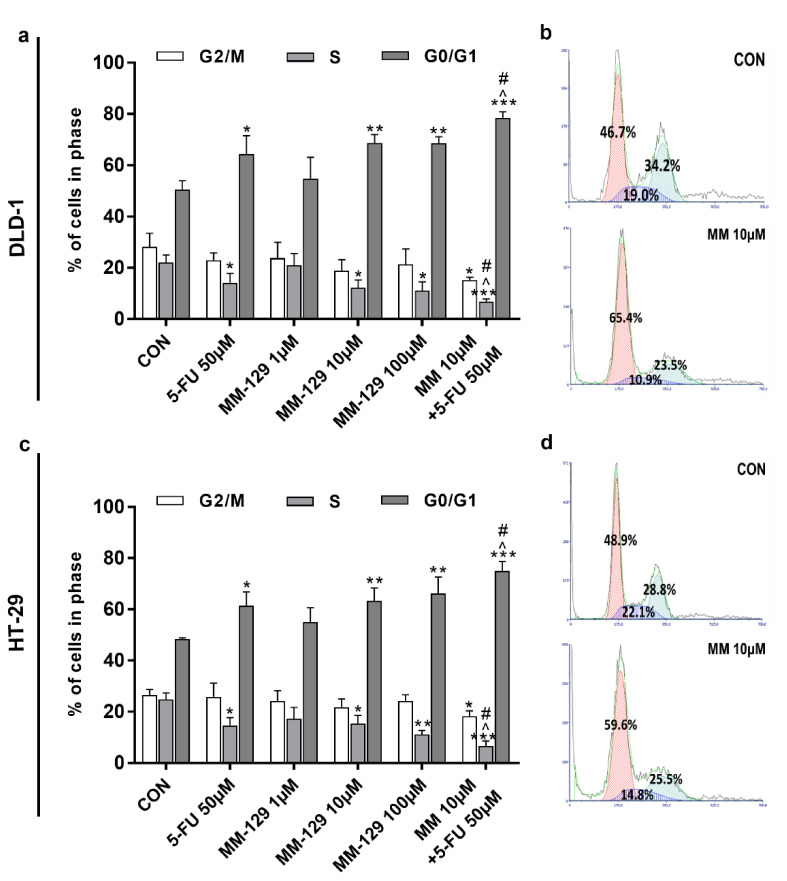
Effect of 5-FU (5-FU 50 µM) and MM-129 (MM 1µM, 10 µM, 100 µM) and their combination on cell cycle progression of DLD-1 (**a**) and HT-29 (**c**). The cells were incubated with 5-FU (5-FU 50 µM), MM-129 (MM 1 µM, 10 µM, 100 µM), and their combination (MM 10 µM + 5-FU 50 µM) for 24 h at 37 °C, and then examined by DNA flow cytometry, as described in the Materials and Methods section. Representative dot-plots presenting the G0/G1 cell-cycle arrest in DLD-1 (**b**), and HT-29 (**d**) cells incubated with MM-129 (MM 10µM); *n* = 3. The results are presented as means ± SDs. * *p* < 0.05, ** *p* < 0.01, *** *p* < 0.001 vs. CON, ^ *p* < 0.05 vs. 5-FU vs. 5-FU, ^#^ *p* < 0.05 vs. MM-129 at dose 10 µM.

The effect of MM-129 and 5-FU on cell cycle distribution was evaluated by flow cytometry. The G1/S check point is crucial for the control of eukaryotic cell proliferation via intracellular and extracellular signals related to the transportation and integration of molecules into the nucleus [14]. The cell cycle distribution of DLD-1 and HT-29 in the control group showed a percentage of cells of 50.4% and 48.4.1% in the G0/G1 phase, and 22.1% and 24.8% in the S phase, respectively (Figure 6a–d). The exposure of DLD-1 to MM-129 (10 µM, 100 µM) or 5-FU brought about a significant increase in the percentage of cells in the G0/G1 phase (68.7%, 68.6%, and 64.3%, respectively), while the proportion of cells in the S phase sharply decreased (12.2%, 11.1%, and 14.1%, respectively). On the other hand, the combination treatment led to a further accumulation of cells in the G0/G1 phase (78.5%) and a reduction in S phase (6.8%) compared to 5-FU and MM-129 alone (Figure 6a,b). Similar changes in HT-29 cells were observed (Figure 6c,d). Our findings show that MM-129 used together with 5-FU potentiated the antimitotic activity of the latter.

To address the possible involvement of the p53 status and CDK2 in MM-129-induced cell cycle arrest, the kinetics of p53 and CDK2 expression were analyzed by RT-PCR, Western blot, and confocal microscopy. p53 is a well-established tumor suppressor protein which prevents tumor growth, and plays a key role in cellular response to cytotoxic stress and cell cycle regulation [36]. RT-PCR analysis showed a marked upregulation of p53 mRNA expression after 24 h treatment with MM-129, as well as with 5-FU in both DLD-1 and HT-29 cells (Figure 2c,d). In addition, simultaneous use of MM-129 and 5-FU even more strongly up-regulated the p53 mRNA, whereby they further increased the population of cells in the G0/G1 phase.

**Figure 7:**
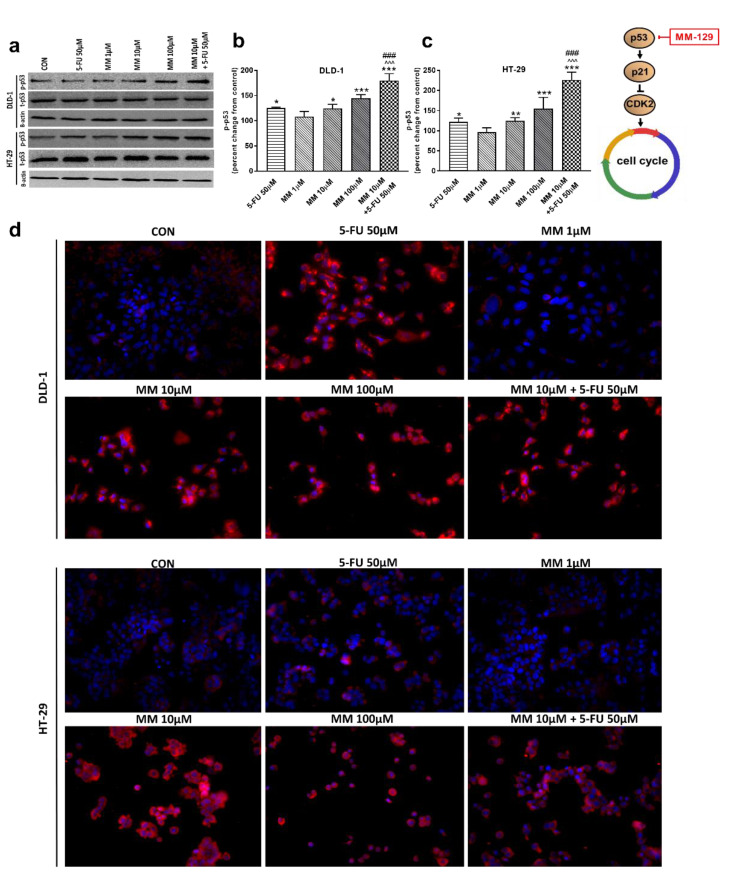
Phosphorylated p53 (p-p53), total p-53 (t-p53), and β-actin expression as determined by Western blot (**a**) and phosphorylated p53 (p-p53) determined by confocal microscopy (**d**) in DLD-1 and HT-29 cells treated with 5-FU (5-FU 50 µM), MM-129 (MM 1 µM, 10 µM, 100 µM), and their combination (MM 10 µM + 5-FU 50 µM) for 24 h. The samples used for electrophoresis consisted of 20 µg of protein from 6 pooled cell extracts. The samples used for electrophoresis consisted of 20 µg of protein from 6 pooled cell extracts from independent experiments (*n* = 6). Band staining was quantified by densitometry (**b**,**c**). The corresponding uncropped blots are shown in Supplementary Figures S7a–c and S8a–c. Cells were incubated with mouse monoclonal antibody against phospho p53 and FITC-conjugated secondary goat polyclonal antibody against mouse (red label). The nuclei were stained with Hoechst 33342 (blue label) (**d**). The results are presented as means ± SDs. * *p* < 0.05, ** *p* < 0.01, *** *p* < 0.001 vs. CON, ^^^ *p* < 0.001 vs. 5-FU, ^###^ *p* < 0.001 vs. MM-129 at dose 10 µM.

Western blot analysis revealed that 5-FU and the novel 1,2,4-triazine derivative at higher doses led to increased expression of p-p53 in both cell lines. In turn, we observed no change in p-p53 expression upon MM-129 use at low concentrations. 5-FU + MM-129 caused the strongest increase in this protein compared with the control cells, 5-FU, and also MM-129 alone. As shown in Figure 7d, in DLD-1 and HT-29 cells treated with 5-FU, MM-129 (at higher doses), and their combination, p-p53 expression was increased together with its translocation to the nuclei. This was particularly pronounced in DLD-1 cells.

**Figure 8:**
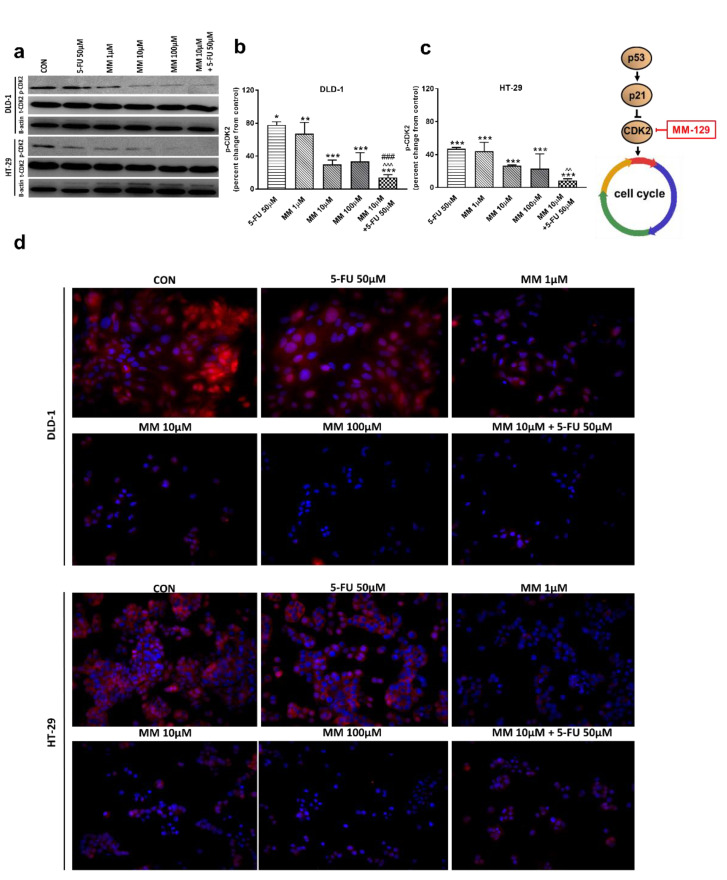
Phosphorylated CDK2 (p-CDK2), total CDK2 (t-CDK2), and β-actin expression as determined by Western blot (**a**) and phosphorylated CDK2 (p-CDK2) determined by confocal microscopy (**d**) in DLD-1 and HT-29 cells treated with 5-FU (5-FU 50 µM), MM-129 (MM 1 µM, 10 µM, 100 µM), and their combination (MM 10 µM + 5-FU 50 µM) for 24 h. The samples used for electrophoresis consisted of 20 µg of protein from 6 pooled cell extracts from independent experiments (*n* = 6). Band staining was quantified by densitometry (**b**,**c**). The corresponding uncropped blots are shown in Supplementary Figures S9a–c and S10a–c. Cells were incubated with rabbit polyclonal antibody against phospho CDK2 and secondary goat polyclonal antibody against rabbit (red label). The nuclei were stained with Hoechst 33342 (blue label) (**d**). The results are presented as means ± SDs. * *p* < 0.05, ** *p* < 0.01, *** *p* < 0.001 vs. CON, ^^ *p* < 0.01 vs. 5-FU, ^^^ *p* < 0.001 vs. 5-FU, ^###^ *p* < 0.001 vs. MM-129 at dose 10 µM.

In the G1 phase, p53 suppresses cell cycle progression through the induction of p21WAF1/CIP1, which inhibits CDK2 and CDK4 [37,38]. Using the Western blot technique, we observed that DLD-1 and HT-29 incubation with 5-FU was the reason for the decreased expression of CDK2 and the further downregulation of this kinase, obtained after the use of MM-129 (Figure 8a–d). Unexpectedly, the strong inhibitory effects of 5-FU noticed in HT-29, determined by Western blot (Figure 8a,c), were not confirmed by confocal microscopy. In turn, RT-PCR, Western blot, and confocal microscopy clearly revealed that simultaneous use of 5-FU with MM-129 significantly decreased CDK2 mRNA (Figure 2e,f) as well as pCDK2 protein expression in both examined lines (Figure 8a–d). Adding MM-129 to 5-FU additionally enhanced these effects by inducing a high level of cell cycle arrest. These results indicate that MM-129 may synergistically augment the antitumor effect of 5-FU by increasing G0/G1 phase arrest. Moreover, a simultaneous use of MM-129 with 5-FU enhances the sensitivity of cancer cells through Akt, mTOR, and CDK2 inhibition.

### 3.4. MM-129 Decreases PD-L1 Expression

**Figure 9:**
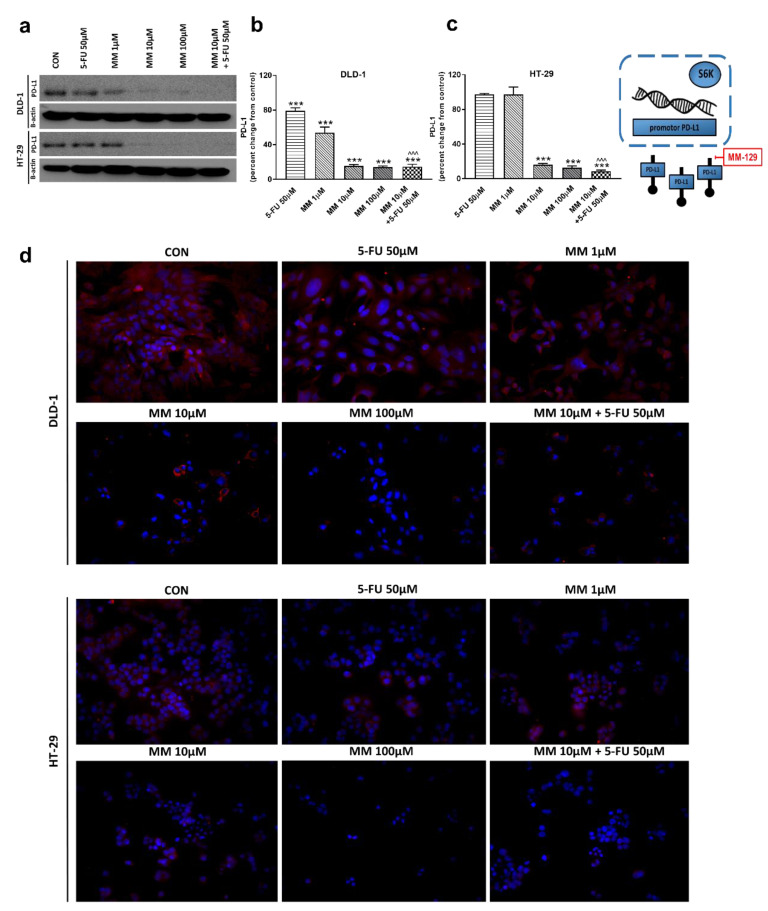
PD-L1 and β-actin expression as determined by Western blot (**a**) and confocal microscopy (**d**) in DLD-1 and HT-29 cells treated with 5-FU (5-FU 50 µM), MM-129 (MM 1 µM, 10 µM, 100 µM), and their combination (MM 10 µM + 5-FU 50 µM) for 24 h. The samples used for electrophoresis consisted of 20 µg of protein from 6 pooled cell extracts from independent experiments (*n* = 6). Band staining was quantified by densitometry (**b**,**c**). The corresponding uncropped blots are shown in Supplementary Figures S11a–c and S12a–c. Cells were incubated with mouse monoclonal antibody against PD-L1 and FITC-conjugated secondary goat polyclonal antibody against mouse (red). The nuclei were stained with Hoechst 33342 (blue) (**d**). The results are presented as means ± SDs. *** *p* < 0.001 vs. CON, ^^^ *p* < 0.001 vs. 5-FU.

The receptor pathway of programmed cell death 1 (PD-1) and its ligand PD-L1 is one of the most-studied immune checkpoints. The primary role of the PD-1 receptor is to inhibit T lymphocyte function, which is observed after its binding to one of the ligands on APC cells. This leads to slower cell metabolism, and thus lymphocyte depletion of their effector functions [39]. Inhibiting the interaction between PD-1 or/and its ligand PD-L1 leads to unblocking T-lymphocyte function, and thus destroying cancer cells. To investigate whether MM-129 affected PD-L1 expression, this protein was determined by RT-PCR, Western blot, and confocal microscopy. The incubation of DLD-1 and HT-29 cells with MM-129 downregulated PD-L1 mRNA expression compared with the control in both DLD-1 and HT-29 cells (Figure 2g,h). Confocal imaging and Western blot confirmed a significant decrease in PD-L1 levels in DLD-1 and HT-29 cells after incubation with MM-129 (Figure 9a–d). 5-FU reduced PD-L1 mRNA (Figure 2g) and protein level (Figure 9a,b) in DLD-1, but not in HT-29 cells (Figure 2h and Figure 9a,c). More detailed investigations of the role of the PD-1/PD-L1 axis in MM-129 antitumor activity will be the subject of our further research using animals with functional immune systems.

However, the addition of MM-129 to 5-FU strongly intensified the effect caused by 5-FU alone in DLD-1 cells (Figure 2g and Figure 9b), and it reduced PD-L1 mRNA and protein level in HT-29 cells (Figure 2h and Figure 9c), in which the inhibitory effect of 5-FU alone was not seen.

## 4. Discussion

In the present study, we showed for the first time that MM-129 (pyrazolo[4,3-e]tetrazolo[4,5-b][1,2,4]triazine sulfonamide) possesses antitumor activity in xenograft mouse models of colon cancer. The mechanistic study revealed that MM-129 not only has the ability to inhibit intracellular pathways promoting tumorigenesis, but also decreases the PD-L1 level, a key element of the immune evasion axis. Moreover, the combined administration of this new compound with 5-FU seems to sensitize the tumor cells to this commonly used chemotherapeutic agent.

Although our previous results documented MM-129-mediated antiproliferative and cytotoxic effects in cultured tumor cells, testing potency using in vivo animal models is generally preferred over in vitro test systems, since animal model assays have the ability to directly measure a product’s functional activity [9,40]. In the present study, high antitumor efficacy was confirmed by a mouse model of xenotransplantation, in which MM-129 caused a significant reduction in tumor volume and mass as early as the second week of therapy. Furthermore, it turned out to be more potent in inhibiting tumor growth compared with 5-FU. It also showed synergistic anticancer effects when used in combination with the latter, especially in the DLD-1 cell line.

Next, we analyzed the possible molecular and biochemical mechanisms responsible for the observed anti-neoplastic activity of MM-129 using DLD-1 and HT-29 colon cancer lines. 5-FU, which remains a mainstay of standard therapy in colon cancers, was used here as a reference drug. Exposure to MM-129 resulted in a decreased expression of p-Akt and p-mTOR in both DLD-1 and HT-29. Stronger downregulation of p-Akt was obtained in DLD-1 cells, which possess a higher expression of Akt than HT-29 cells (Figure 3a). Thus, we presume that a higher expression of Akt, which is an MM-129 target protein, may explain the potent antitumor activity of this compound observed in DLD-1 xenografts. This is in accordance with in vivo results, in which we found a more effective tumor growth reduction in DLD-1 xenografts. Previously, Chen and colleagues also reported that the inhibition of PI3K/Akt/mTOR signaling results in decreased CCSCs proliferation and leads to the suppression of xenograft tumor growth [41]. Moreover, Kim et al. provided evidence that over-activation of Akt is crucial for the acquisition of resistance to 5-fluorouracil in 5-FU-resistant human colon cancer cells (SNU-C5/5-FU cells). They found that SNU-C5/5-FU cells had higher levels of phospho-Akt, phospho-mTOR, nuclear β-catenin, COX-2, and survivin, and a lower level of E-cadherin compared to parental SNU-C5/WT cells. In addition, treatment with LY294002, a PI3K kinase inhibitor, resulted in the enhancement of apoptosis in SNU-C5/5-FU following 5-FU treatment [32]. Here, we observed no effect on p-Akt expression in DLD-1 and HT-29 when 5-FU was used alone. In turn, a simultaneous use of MM-129 with 5-FU significantly decreased p-AKT expression levels compared with the control and 5-FU alone, but not with MM-129 alone.

Blocking the PI3K/AKT pathway was sufficient to cause cell cycle arrest, manifested as an increased population of cells in the G0/G1 phase. The induction of apoptosis also seems likely, because the existing data suggested that the activation of Akt allows SNU-C5/5-FU cells to avoid apoptosis through a direct interaction of caspase-3 with survivin [32]. Consequently, our results suggested that the inhibition of Akt activation may enhance the sensitivity to 5-FU in both DLD-1 and HT-29 cells. These findings are in agreement with our present and also previous study, in which we reported that MM-129 effectively inhibits tumor development in the zebrafish embryo xenograft model, and it shows a markedly synergistic anticancer effect when used in combination with 5-FU [9].

Cancer cells are often characterized by cell cycle abnormalities, which lead to unregulated proliferation. In the present study, cell cycle analysis showed that the treatment of DLD-1 and HT-29 cells with MM-129 brought an increase in the percentage of cells in the G0/G1 phase, with a concurrent decrease of cells in the S phase. These observations are consistent with findings from other studies. Roscovitine, which has a similar chemical structure to MM-129, also arrested cancer cells RPMI 8226 (human multiple myeloma cells) in G1 phase [42]. Liu et al. also presented that lung carcinoma cells A549 treated with WZB117 (inhibitor of glucose transporter 1) resulted in approximately 23% more cells in the G0/G1 phase and approximately 30% fewer cells in the S-phase [43]. Moreover, our previous study reported that DLD-1 and HT-29 cells treated with LFM-A13 show an S phase fraction reduction and a slight increase in the phase G0/G1 [26]. We obtained similar results when cells were treated with 5-FU. The available data indicate that 5-FU induces a marked increase in the relative cell numbers in G1 and possibly early S fractions; therefore, it arrested colon cancer cells SW620 in the S phase, and oral cancer cells HSC-4 in G1/S phase, while gastric cancer cells AGS were arrested in the G0/G1 phase [44,45,46]. After the combined treatment of MM-129 with 5-FU, the specific G0/G1 phase accumulation was markedly increased, which strongly supports the ability of MM-129 to enhance the effects of 5-FU. As a result, the cell cycle arrest in the G0/G1 phase was probably one of the underlying mechanisms of synergistic interactions between MM-129 and 5-FU.

To determine whether the expression levels of cell-cycle-related proteins were changed, the expression of p53 and CKD2 using real-time RT-PCR, Western blot technique, and confocal microscopy was analyzed. Once p53 is induced, a host of target genes are then transcriptionally activated, including p21, GADD45 (the growth arrest and DNA damage-inducible 45 protein), bax, and bcl-2. The induction of p21, in turn, leads to cell cycle arrest at the G1 checkpoint [47]. The inhibition of CDK2 by p21 blocks Rb protein (pRb) phosphorylation, promotes pRb binding to E2F1 (transcription factor), and promotes transcription silencing of E2F1 targets critical for DNA replication and cell-cycle progression [36,38]. As expected, the remarkable up-regulation of p-p53, and a concomitant down-regulation of p-CKD2, were observed after 24 h treatment with MM-129 and 5-FU in both cell lines. These changes were probably responsible for the cell-cycle arrest. Our results are in line with Nazim et al., who reported that 5-fluorouracil enhances p53 expression in A549 lung adenocarcinoma cells and induces apoptosis via the p53-dependent pathway [48]. A stronger effect after simultaneous administration of MM-129 with 5-FU was obtained. The results indicate that MM-129 may act as a chemosensitizer and enhance the sensitivity of colon cancer cells to 5-FU.

The phenomenon of cancer cells avoiding the immune response discovered in recent years and the unprecedented results of immune checkpoint inhibitors have become the basis of development of a new, groundbreaking direction in cancer treatment, which is immuno-oncology. Restoring proper immune system functioning has proved to be an effective strategy for fighting cancer. Due to the similarity of the structure of MM-129 to roscovitine, which attenuates tumor PD-L1 expression and promotes antitumor immunity, we decided to check whether a new 1,2,4-triazine derivative can also promote an immunological anti-tumor response [49]. We found that MM-129, in a dose-dependent manner, significantly decreased PD-L1 expression in both cell lines. No effects were observed after the exposure to 5-FU alone in HT-29 cells, whereas the combination of MM-129 and 5-FU was synergistic in lowering PD-L1 level in both examined lines. Our results are consistent with the observations of Cortez and colleagues, who documented that the induction of p53 leads to the downregulation of PD-L1 [50]. Moreover, Lastwika et al. reported that the PI3K/Akt/mTOR pathway participates in the development of tumor cell immunoresistance. It was proved that the membrane PD-L1 expression in human lung cancer cells is significantly associated with mTOR kinase activation, and the activation of the Akt/mTOR axis promotes immune escape via enhanced PD-L1 expression [51]. Still, the use of mTOR inhibitor in combination with the PD-1 antibody in the mouse lung cancer model resulted in a significant inhibition of tumor growth, an increase in tumor-infiltrating lymphocytes (TILs) number, and a significant reduction in Tregs number [51]. Additional PTEN (tumor suppressor phosphatase and tensin homolog) gene mutation results in the loss of ability to inhibit the PI3K/Akt/mTOR/S6K pathway, which leads to increased PD-L1 expression. The gene that encodes PTEN often mutates in breast, ovarian, kidney, glioblastoma, melanoma, and lung cancers. The most recent data from clinical and preclinical studies indicate that PI3K/Akt/mTOR inhibition may have a double benefit. On the one hand, it may limit tumor growth by inhibiting cell proliferation, migration, and survival. On the other hand, it may increase immunological anti-tumor control by blocking the activation of immunosuppressive pathways and strengthening internal immunity mechanisms [52]. An additional study will be needed to explain the potential involvement of MM-129 in the activation of the body’s natural immune system. These issues will be the subject of our future research.

## 5. Conclusions

The search for compounds with alternative mechanisms of action in relation to known anticancer drugs is the leading research aim of modern science and the pharmaceutical industry. Multi-targeting of oncoproteins by a single drug molecule represents an innovative, efficient, and logical approach in relation to drug combinations. Our results provide unprecedented evidence that MM-129 has the ability to inhibit intracellular pathways promoting tumorigenesis with a simultaneous reduction of PD-L1 expression, a key element of the cancer immune escape axis. An additional combination therapy with MM-129 and 5-FU significantly intensified the antitumorigenic effects of 5-FU by increasing the sensitivity of cancer cells. Moreover, recent reports also indicate the participation of the PD-1/PD-L1 pathway in the origination of tumor cell resistance, which further reinforces the conviction that the search for drugs with multidirectional effects can effectively inhibit the neoplastic process [53,54]. This preclinical research provides a basis for future pre-IND studies and clinical development of MM-129 as a new agent against colon cancer with a multi-targeting mechanism of action.

## Data Availability

Not applicable.

## Supplementary Materials

The following are available online at https://www.mdpi.com/article/10.3390/cancers13133203/s1, Figure S1: Uncropped Western blot image of Akt from DLD-1 cells, Figure S2: Uncropped Western blot image of Akt from HT-29 cells, Figure S3: Uncropped Western blot image of mTOR from DLD-1 cells, Figure S4: Uncropped Western blot image of mTOR from HT-29 cells, Figure S5: Uncropped Western blot image of ERK1/2 from DLD-1 cells, Figure S6: Uncropped Western blot image of ERK1/2 from HT-29 cells, Figure S7: Uncropped Western blot image of p53 from DLD-1 cells, Figure S8: Uncropped Western blot image of p53 from HT-29 cells, Figure S9: Uncropped Western blot image of CDK2 from DLD-1 cells, Figure S10: Uncropped Western blot image of CDK2 from HT-29 cells, Figure S11: Uncropped Western blot image of PD-L1 from DLD-1 cells, Figure S12: Uncropped Western blot image of PD-L1 from HT-29 cells.
