# Supplementary material for: MM-129 as a Novel Inhibitor Targeting PI3K/AKT/mTOR and PD-L1 in Colorectal Cancer
Source: Cancers (Basel). 2021 Jun 26;13(13):3203. doi: 10.3390/cancers13133203 (PMC8268553; doi:10.3390/cancers13133203)
Supplement: Supplementary file 1 [file cancers-13-03203-s001.zip › cancers-1179184-suppl-final.pdf]

cancers

MDPI

Article

MM-129 as a Novel Inhibitor Targeting PI3K/AKT/mTOR and PD-L1 in Colorectal Cancer

Justyna Magdalena Hermanowicz, Krystyna Pawlak, Beata Sieklucka, Robert Czarnomysy, Iwona Kwiatkowska, Adam Kazberuk, Arkadiusz Surazynski, Mariusz Mojzych and Dariusz Pawlak

Citation: Hermanowicz, J.M.; Pawlak, K.; Sieklucka, B.; Czarnomysy, R.; Kwiatkowska, I.; Kazberuk, A.; Surazynski, A.; Mojzych, M.; Pawlak, D. MM-129 as a Novel Inhibitor Targeting PI3K/AKT/mTOR and PD-L1 in Colorectal Cancer. *Cancers* **2021**, *13*, 3203. https://doi.org/10.3390/cancers13133203

Academic Editor(s): Alessandro Cama

Received: 26 March 2021
Accepted: 22 June 2021
Published: date

Publisher's Note: MDPI stays neutral with regard to jurisdictional claims in published maps and institutional affiliations.

Copyright: © 2021 by the authors. Submitted for possible open access publication under the terms and conditions of the Creative Commons Attribution (CC BY) license (http://creativecommons.org/licenses/by/4.0/).

Supplementary Materials:

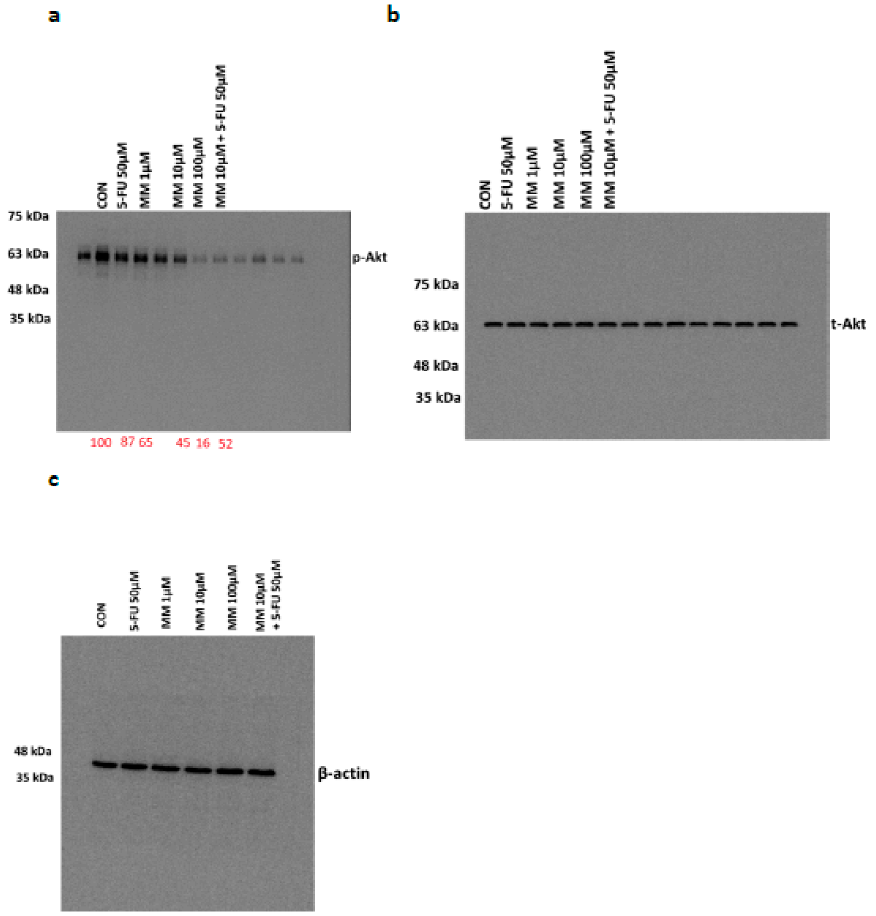

Figure S1. Uncropped Western blot image of phosphorylated Akt (p-Akt) (a) total Akt (t-Akt) (b) and β-actin (c) from DLD-1 cells treated with 5-FU (5-FU 50 µM) and MM-129 (MM 1µM, 10 µM, 100 µM) and their combination (MM 10 µM + 5-FU 50 µM) for 24 h. The corresponding cropped blots are shown in Figure 3a of the main text.

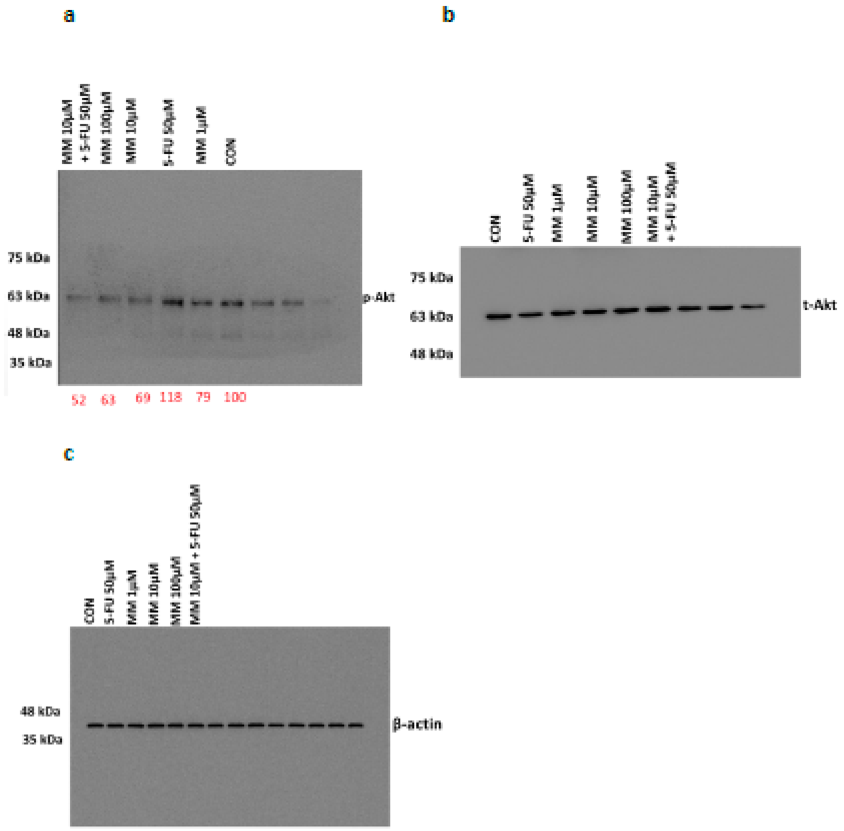

Figure S2. Uncropped Western blot image of phosphorylated Akt (p-Akt) (**a**) total Akt (t-Akt) (**b**) and β-actin (**c**) from HT-29 cells treated with 5-FU (5-FU 50 µM) and MM-129 (MM 1µM, 10 µM, 100 µM) and their combination (MM 10 µM + 5-FU 50 µM) for 24 h. The corresponding cropped blots are shown in Figure 3a of the main text.

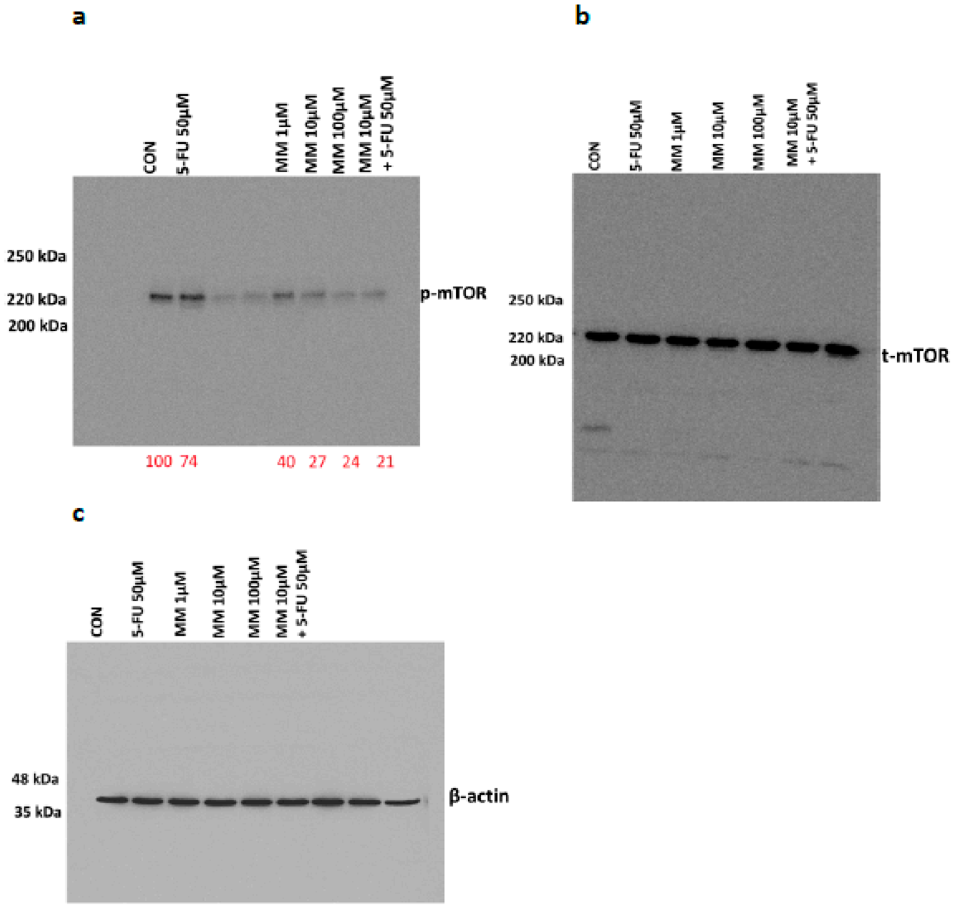

Figure S3. Uncropped Western blot image of phosphorylated mTOR (p-mTOR) (a) total mTOR (t-mTOR) (b) and β-actin (c) from DLD-1 cells treated with 5-FU (5-FU 50 µM) and MM-129 (MM 1µM, 10 µM, 100 µM) and their combination (MM 10 µM + 5-FU 50 µM) for 24 h. The corresponding cropped blots are shown in Figure 4a of the main text.

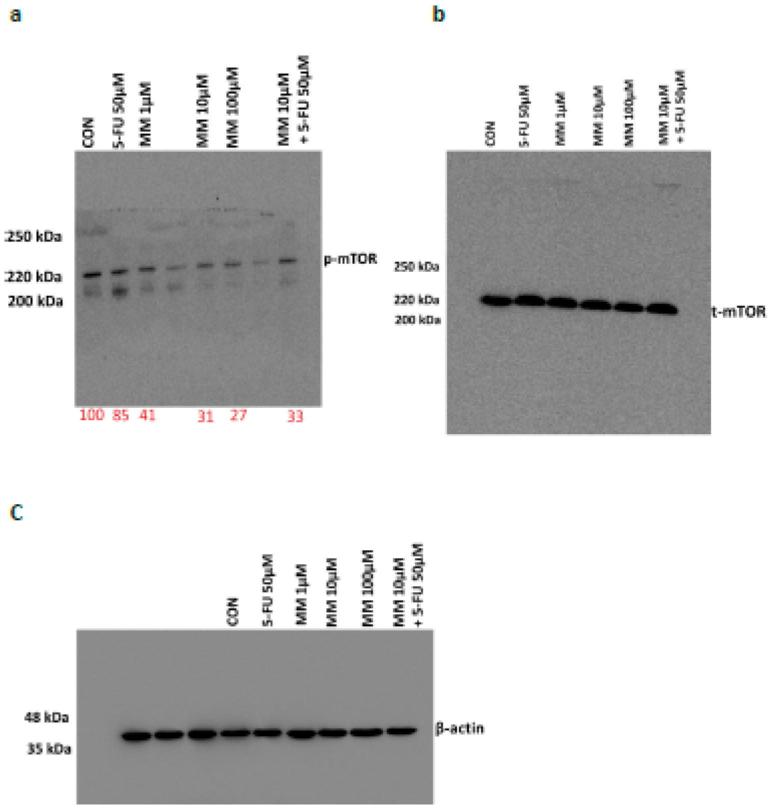

Figure S4. Uncropped Western blot image of phosphorylated mTOR (p-mTOR) (a) total mTOR (t-mTOR) (b) and β-actin (c) from HT-29 cells treated with 5-FU (5-FU 50 μM) and MM-129 (MM 1μM, 10 μM, 100 μM) and their combination (MM 10 μM + 5-FU 50 μM) for 24 h. The corresponding cropped blots are shown in Figure 4a of the main text.

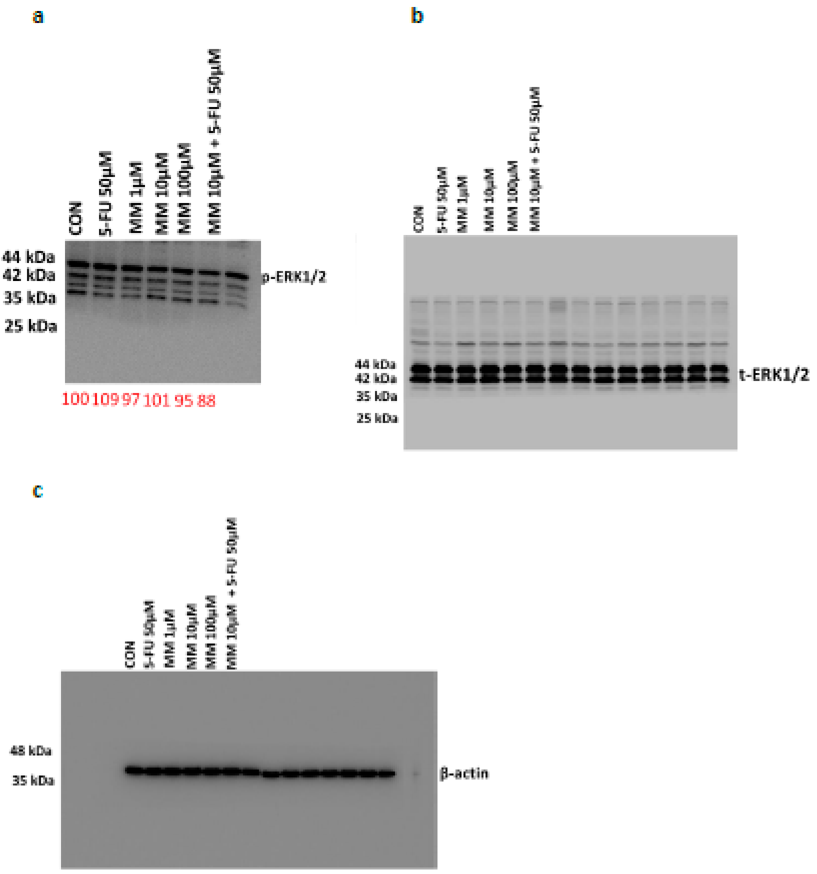

Figure S5. Uncropped Western blot image of phosphorylated ERK1/2 (p-ERK1/2) (a) total ERK1/2 (t-ERK1/2) (b) and β-actin (c) from DLD-1 cells treated with 5-FU (5-FU 50 μM) and MM-129 (MM 1μM, 10 μM, 100 μM) and their combination (MM 10 μM + 5-FU 50 μM) for 24 h. The corresponding cropped blots are shown in Figure 5a of the main text.

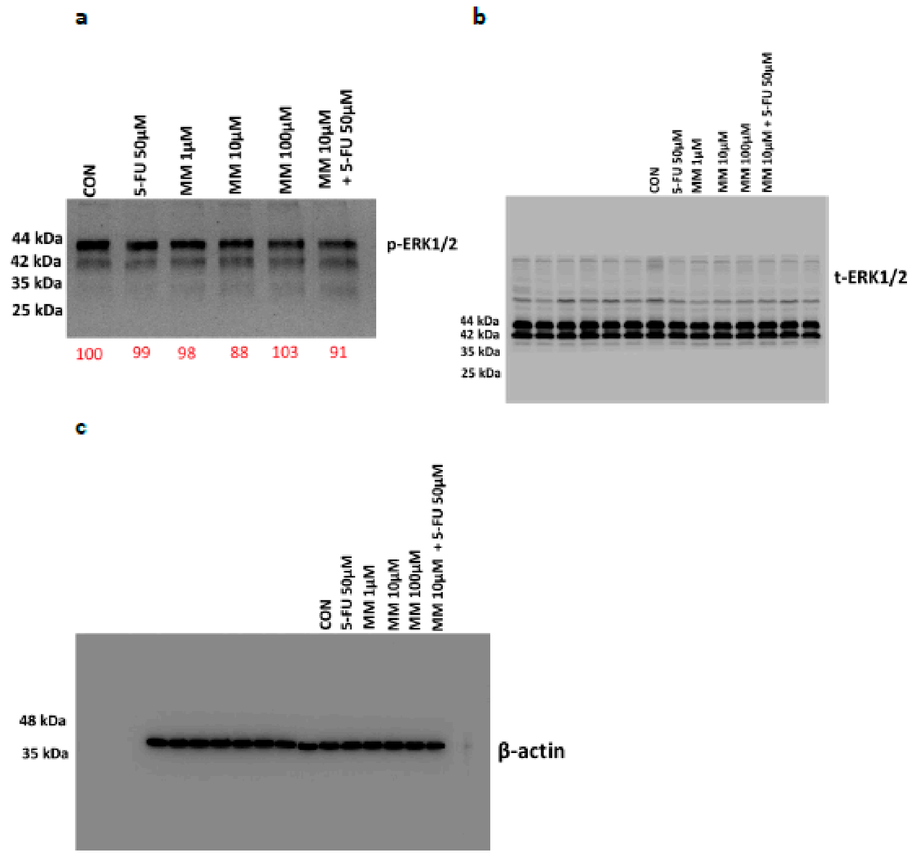

Figure S6. Uncropped Western blot image of phosphorylated ERK1/2 (p-ERK1/2) (a) total ERK1/2 (t-ERK1/2) (b) and β-actin (c) from HT-29 cells treated with 5-FU (5-FU 50 μM) and MM-129 (MM 1μM, 10 μM, 100 μM) and their combination (MM 10 μM + 5-FU 50 μM) for 24 h. The corresponding cropped blots are shown in Figure 5a of the main text.

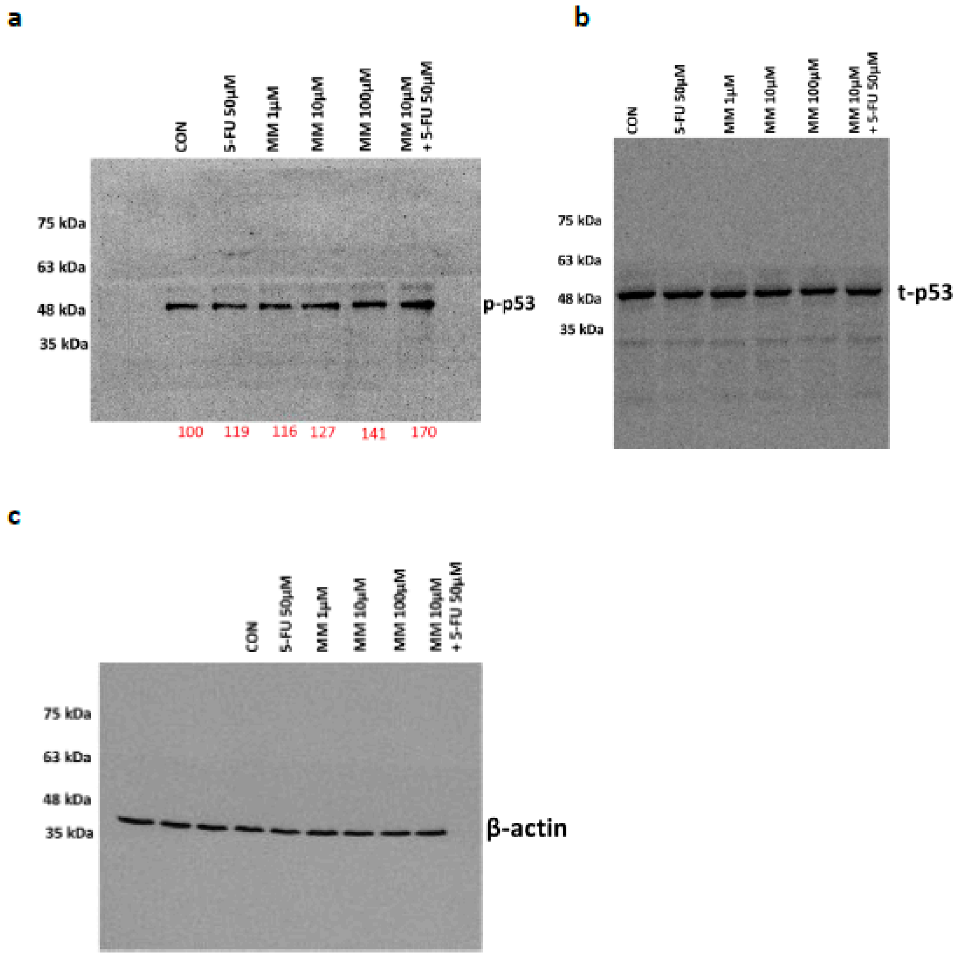

Figure S7. Uncropped Western blot image of phosphorylated p53 (p-p53) (a), total p-53 (t-p53) (b) and β-actin (c) from DLD-1 cells treated with 5-FU (5-FU 50 µM) and MM-129 (MM 1µM, 10 µM, 100 µM) and their combination (MM 10 µM + 5-FU 50 µM) for 24 h. The corresponding cropped blots are shown in Figure 7a of the main text.

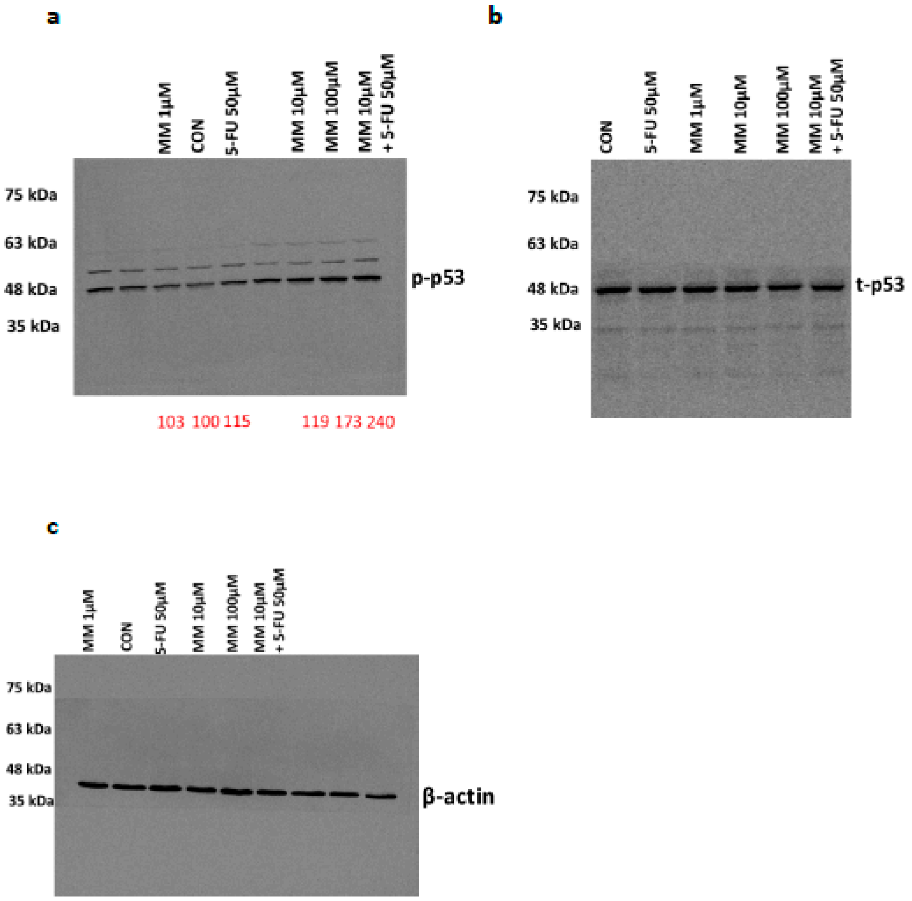

Figure S8. Uncropped Western blot image of phosphorylated p53 (p-p53) (a), total p-53 (t-p53) (b) and β-actin (c) from HT-29 cells treated with 5-FU (5-FU 50 µM) and MM-129 (MM 1µM, 10 µM, 100 µM) and their combination (MM 10 µM + 5-FU 50 µM) for 24 h. The corresponding cropped blots are shown in Figure 7a of the main text.

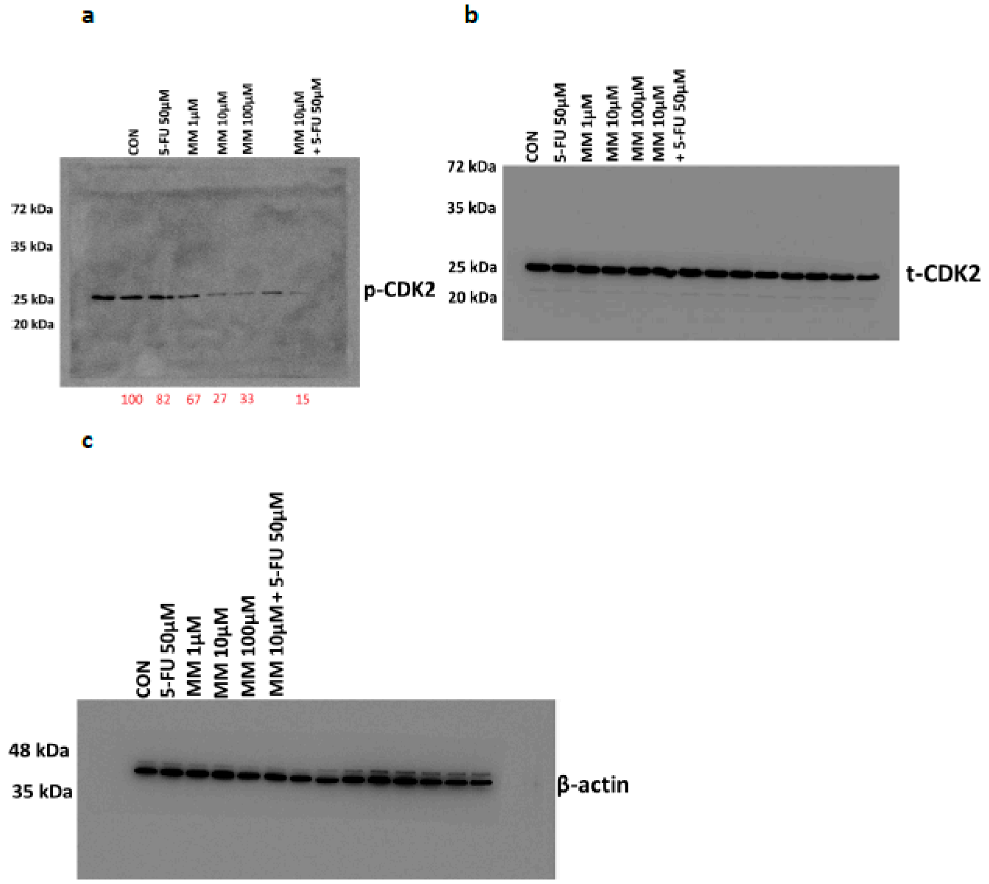

Figure S9. Uncropped Western blot image of phosphorylated CDK2 (p-CDK2) (a), total CDK2 (t-CDK2) (b) and β-actin (c) from DLD-1 cells treated with 5-FU (5-FU 50 µM) and MM-129 (MM 1µM, 10 µM, 100 µM) and their combination (MM 10 µM + 5-FU 50 µM) for 24 h. The corresponding cropped blots are shown in Figure 8a of the main text.

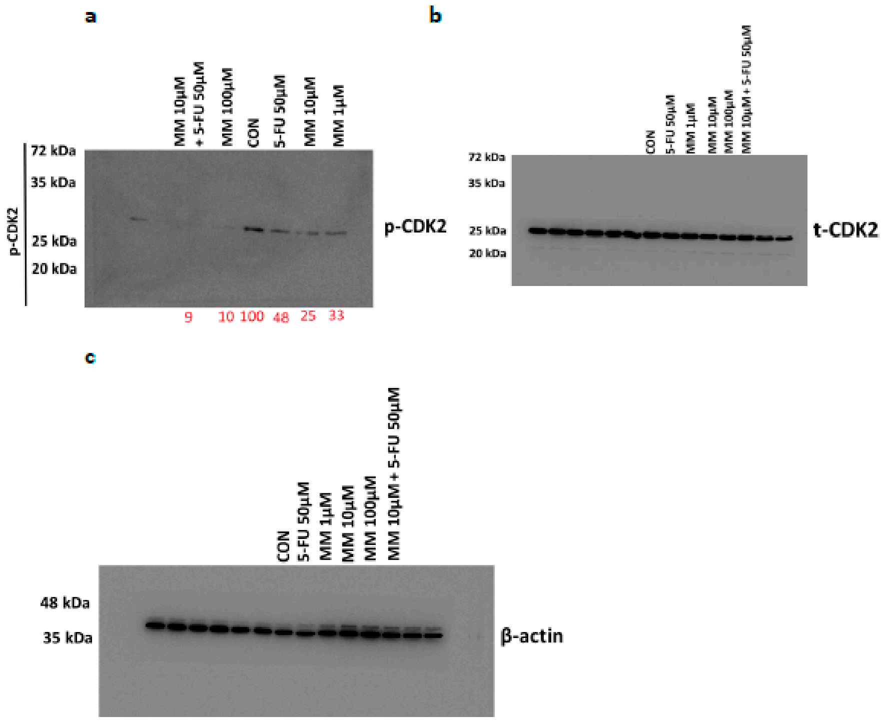

Figure S10. Uncropped Western blot image of phosphorylated CDK2 (p-CDK2) (a), total CDK2 (t-CDK2) (b) and β-actin (c) from HT-29 cells treated with 5-FU (5-FU 50 µM) and MM-129 (MM 1µM, 10 µM, 100 µM) and their combination (MM 10 µM + 5-FU 50 µM) for 24 h. The corresponding cropped blots are shown in Figure 8a of the main text.

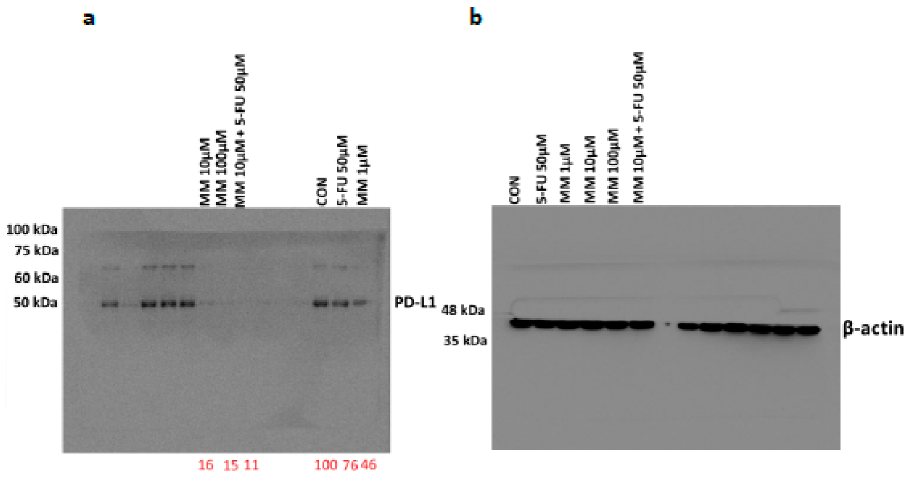

Figure S11. Uncropped Western blot image of phosphorylated PD-L1 (a) and β-actin (b) from DLD-1 cells treated with 5-FU (5-FU 50 µM) and MM-129 (MM 1µM, 10 µM, 100 µM) and their combination (MM 10 µM + 5-FU 50 µM) for 24 h. The corresponding cropped blots are shown in Figure 9a of the main text.

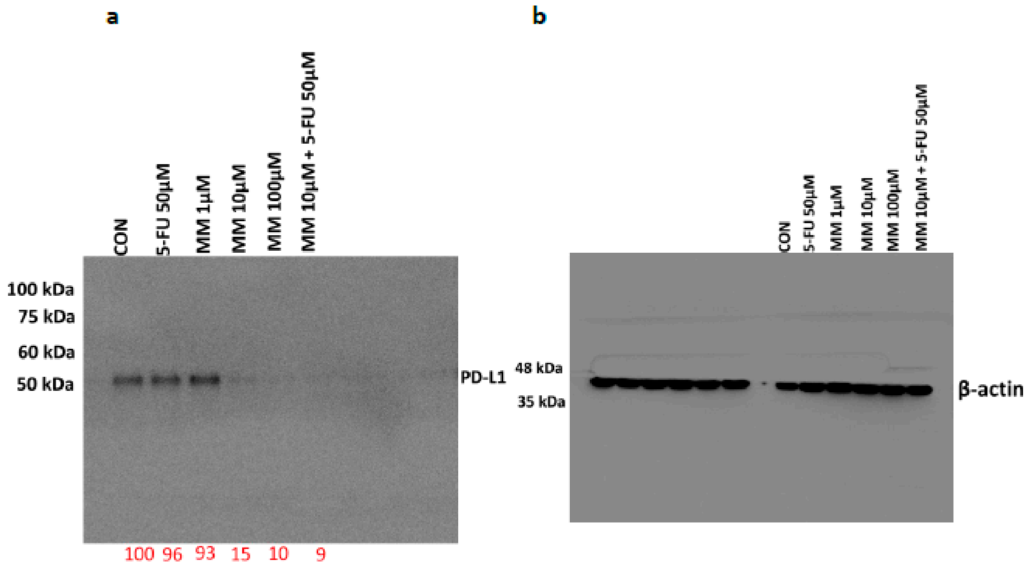

Figure S12. Uncropped Western blot image of phosphorylated PD-L1 (a) and β-actin (b) from HT-29 cells treated with 5-FU (5-FU 50 µM) and MM-129 (MM 1µM, 10 µM, 100 µM) and their combination (MM 10 µM + 5-FU 50 µM) for 24 h. The corresponding cropped blots are shown in Figure 9a of the main text.
